# Effect of third- and fourth-line systemic therapies for metastatic renal cell carcinoma

**DOI:** 10.1038/s41598-019-51305-7

**Published:** 2019-10-29

**Authors:** Sei Naito, Osamu Ichiyanagi, Tomoyuki Kato, Hidenori Kanno, Takafumi Narisawa, Masayuki Kurokawa, Masaki Ushijima, Michinobu Ozawa, Mayu Yagi, Yuta Kurota, Hiroki Fukuhara, Atsushi Yamagishi, Toshihiko Sakurai, Hayato Nishida, Hisashi Kawazoe, Takuya Yamanobe, Norihiko Tsuchiya

**Affiliations:** Department of Urology, Yamagata University Faculty of Medicine, Iida-nishi 2-2-2, Yamagata, 990-9585 Japan

**Keywords:** Outcomes research, Molecular medicine, Renal cell carcinoma

## Abstract

Data on the outcomes of third- or fourth-line therapy for metastatic renal cell carcinoma (mRCC) are limited. The aim of our study was to evaluate the efficacy of therapy beyond the second line. We retrospectively analysed data of mRCC patients who underwent systemic therapy at Yamagata University Hospital. The best objective response (BOR), response rate (RR), and progression-free survival (PFS) were assessed for each line of treatment. To investigate the correlation between overall survival (OS) and the number of treatment lines during a patient’s lifetime, the median OS was assessed using univariate and multivariate analyses. In the first-, second-, and third-line therapies, approximately 20% of patients had long PFS of >15 months. In targeted treatments beyond the third line, only one treatment suppressed disease progression for >10 months. Among patients who died during the follow-up period, those treated with triple and quadruple lines had similar OS (42.5 months vs. 48.4 months, respectively). Multivariate analysis showed that patients with triple or more lines of therapy had better OS; however, quadruple or more lines of therapy was not an independent prognostic factor. We concluded that third-line systemic therapy could improve OS; however, fourth-line therapy could not.

## Introduction

Systemic therapy for patients with metastatic renal cell carcinoma (mRCC) primarily consisted of cytokine therapies, including interferon-alpha and interleukin-2, until the early 2000s^1,2^. Introduction of two types of targeted treatment (TT), anti-vascular endothelial growth factor (αVEGF) agents and mammalian target of rapamycin inhibitors (mTORIs), after the late 2000s has changed the therapeutic strategies for mRCC and improved its prognosis^3–6^. More recently, immune-oncologic treatment (IoT) has been evoking further change for mRCC treatment. Nivolumab has been demonstrated the priority as sequential therapy, when compared to the mTORI, everolimus^7^. In addition, the combination regimens of IoT and/or αVEGF agent were established as first-line systemic therapy for mRCC^8–10^.

Although the number of novel therapies has increased, the effects of third- and fourth-line sequential therapies remain controversial^4^. This is, because the majority of patients evaluated in clinical randomised controlled trials (RCTs) received the first- or second-line therapy. Nevertheless, retrospective studies have suggested the efficacy of third-line therapy for improving overall survival (OS). According to the International mRCC Database Consortium (IMDC) cohort, which included 1012 patients with third-line TT, the median OS from initiation of third-line therapy was 12.4 months. This was longer than the median OS from the termination of second-line therapy, in patients without third-line therapy^11^. A Swiss single centre study also showed that the median OS in patients with triple or more lines of treatment was longer than that of those with single- or double-line treatments (43.8 vs. 17.6 months, respectively)^12^.

With four or more lines, however, evidence (except for two reports) is lacking. The Swiss report with 13 patients who underwent fourth-line therapy showed a response rate (RR) of 15.4%, lower than that with previous lines^12^. A Japanese report with 12 patients showed a progression-free survival (PFS) of 2.5 months. Both reports failed to describe the OS in the fourth-line patients^13^. Hence, we retrospectively evaluated mRCC patients, focusing on third and fourth lines.

## Results

### Characteristics of the 143 mRCC patients

A total of 143 patients underwent systemic therapy at Yamagata University Hospital. The median OS in this cohort was estimated to be 34.8 months (95% confidential interval [95% CI]: 25.0–46.8 months). The median age at first systemic therapy was 65.6 years (range: 34.8–83.4 years). A total of 17 (14.0%), 82 (67.8%), and 22 (18.2%) patients were classified into the favourable, intermediate, and poor risk groups, respectively, according to the Memorial Sloan Kettering Cancer Centre (MSKCC) criteria. At the time of the database lock, 40 (28.0%) patients were alive and 103 (72.0%) were deceased (Table 1).

**Table 1:** Characteristics of all patients and patients who died during the follow-up period from the date of initial systemic therapy.

Characteristics	All patients	Patients who died
All	143	103
Median overall survival (95% CI)	34.8 months (25.0–46.8)	17.7 months (14.2–29.2)
**Age**		
median (range)	65.6 years (34.8–83.4)	65.9 years (34.8–83.4)
**Sex**		
male (%)	109 (77.2)	79 (81.1)
female (%)	34 (23.8)	24 (21.9)
**Outcome**		
alive (%)	40 (28.0)	0
death due to renal cell carcinoma (%)	96 (67.1)	96 (93.2)
non-renal cell carcinoma-related death (%)	7 (4.9)	7 (6.8)
**MSKCC criteria**		
favourable (%)	17 (14.0)	5 (5.7)
intermediate (%)	82 (67.8)	61 (70.1)
poor (%)	22 (18.2)	21 (24.1)
unknown	22	16
**IMDC criteria**		
favourable (%)	12 (11.3)	4 (5.4)
intermediate (%)	73 (68.9)	50 (67.6)
poor (%)	21 (19.8)	20 (27.0)
unknown	37	29
**Cytokine**		
Yes (%)	43 (30.1)	29 (28.2)
No (%)	100 (69.9)	74 (71.8)

Abbreviations: 95% CI; 95% confidential interval, MSKCC; Memorial Sloan Kettering Cancer Center, IMDC; International Metastatic Renal Cell Carcinoma Database Consortium.

**Figure 1:**
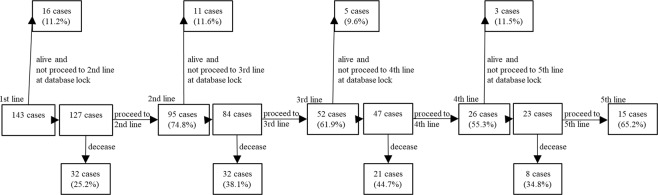
Systemic review of patient statuses.

Among 127 patients, after the first-line treatment, 95 (74.8%) proceeded to the second line. Patients who proceeded to third-line therapy from second-line therapy, to fourth-line therapy from third-line therapy, and to fifth-line therapy from fourth-line therapy were 52 (61.9%), 26 (55.3%), and 15 (65.2%), respectively. Overall, 44.8% of 116 patients who were not in first- or second-line therapy at database lock reached third-line therapy, 23.4% reached fourth-line therapy, and 13.9% reached fifth-line therapy (Fig. 1). Subsequently, 5, 3, and 1 patients proceeded to the sixth-, seventh-, and eighth-line treatments, respectively (Supplementary Table 1).

The first-, second-, third-, and fourth line rates of αVEGF/mTORI were 88.1%/6.3%, 80.0%/12.6%, 46.2%/28.8%, and 53.8%/26.9%, respectively. More patients underwent αVEGF in the first and second lines, and mTORI in the third and fourth lines (Supplementary Table 1).

### RR, DCR, and PFS for each line

**Figure 2:**
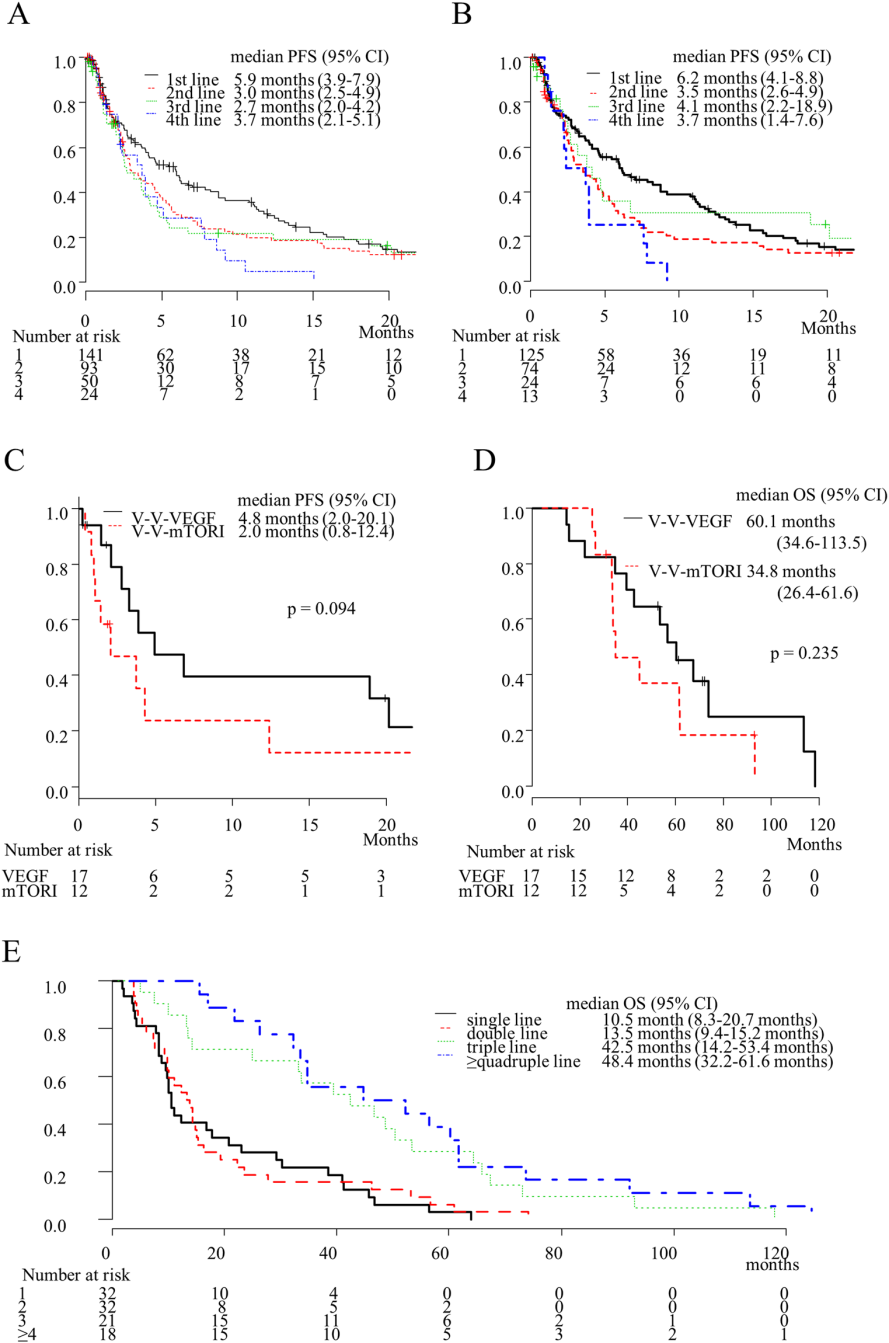
Progression-free survival (PFS) for each treatment line in all patients (**A**) and in patients with targeted therapy. (**B**) PFS during third-line (**C**) and overall survival (OS) (**D**) in patients with a sequence of anti-vascular endothelial growth factor (αVEGF)-αVEGF-αVEGF and a sequence of αVEGF-αVEGF-mammalian target of rapamycin inhibitor (mTORI). OS for each treatment line administered during entire patients’ lifetimes in those patients who died during the follow-up period (**E**).

RRs in the first-, second- third-, and fourth-line therapies were 14.7%, 8.4%, 7.7%, and 3.8%, respectively. A comparatively low rate of treatments beyond the third-line therapy showed clinical response. Disease control rates (DCRs) in the first-, second- third-, and fourth-line therapies were 45.5%, 28.4%, 26.9%, and 23.1%, respectively. There was little difference among the lines except first-line therapy with regard to DCR (Table 2). Figure 2A shows the PFS for each line. The median PFS in the first-, second-, third-, and fourth-line therapies was 5.9, 3.0, 2.7, and 3.7 months, respectively. PFS with the first-line therapy was relatively longer than that with subsequent lines. However, PFS with the second-, third-, and fourth-line therapies was nearly the same in duration.

**Table 2:** Best objective response for each line.

	N	CR (%)	PR (%)	SD (%)	PD (%)	Unavailable
1st line	143	1 (0.7)	20 (14.0)	44 (30.8)	53 (37.1)	25
αVEGF	126	1 (0.8)	19 (15.1)	40 (31.7)	44 (34.9)	22
mTORI	9	0	0	0	6 (66.7)	3
IoT	1	0	1 (100)	0	0	0
Other	7	0	0	4 (57.1)	3 (42.9)	0
2nd line	95	2 (2.1)	6 (6.3)	19 (20.0)	50 (52.6)	18
αVEGF	76	1 (1.3)	5 (6.6)	15 (19.7)	40 (52.6)	15
mTORI	12	0	1 (8.3)	3 (25.0)	6 (50.0)	2
IoT	2	0	0	0	1 (50.0)	1
Other	5	1 (20.0)	0	1 (20.0)	3 (60.0)	0
3rd line	52	0	4 (7.7)	10 (19.2)	26 (50.0)	12
αVEGF	24	0	3 (12.5)	5 (20.8)	9 (37.5)	7
mTORI	15	0	0	2 (13.3)	11 (73.3)	2
IoT	9	0	1 (11.1)	2 (22.2)	4 (44.4)	2
Other	4	0	0	1 (25.0)	2 (50.0)	1
4th line	26	0	1 (3.8)	5 (19.2)	15 (57.7)	5
αVEGF	14	0	1 (7.1)	2 (14.3)	9 (64.3)	2
mTORI	7	0	0	2 (28.6)	3 (42.9)	2
IoT	2	0	0	1 (50.0)	1 (50.0)	0
Other	3	0	0	0	2 (66.7)	1
5th line	15	0	2 (13.3)	1 (6.7)	11 (73.3)	1
αVEGF	5	0	0	1 (20.0)	3 (60.0)	1
mTORI	3	0	0	0	3 (100)	0
IoT	4	0	2 (50.0)	0	2 (50.0)	0
Other	3	0	0	0	3 (100)	0
6th line	5	0	0	1 (20.0)	3 (60.0)	1
αVEGF	5	0	0	1 (20.0)	3 (60.0)	1
7th line	3	0	1 (33.3)	0	2 (66.7)	0
αVEGF	1	0	0	0	1 (100)	0
IoT	1	0	1 (100)	0	0	0
Other	1	0	0	0	1 (100)	0
8th line	1	0	0	0	1 (100)	0
αVEGF	1	0	0	0	1 (100)	0

Abbreviations: N; number, CR; complete response, PR; partial response, SD; stable disease, PD; progression disease, αVEGF; anti-vascular endothelial growth factor; mTORI: mammalian target of rapmycin inhibitor; IoT: immune-oncologic treatment.

With regard to TT, RRs with the first-, second-, third-, and fourth-line therapies were 15.9%, 8.1%, 12.5%, and 7.1%, respectively. In TT beyond third-line therapy, only one treatment sorafenib showed clinical response. The DCRs were 47.6%, 27.6%, 33.3%, and 21.4%, respectively (Table 2). The median PFS with the first-, second-, third-, and fourth-line therapies was 5.9, 3.4, 3.2, and 3.7 months, respectively (Fig. 2B). No treatment exceeded 10 months efficacy beyond the third-line therapy except sixth-line axitinib therapy in one patient (Supplementary Table 2). This patient underwent three lines of αVEGF before axitinib; sunitinib was the first-line therapy (at 11.0 months treatment was terminated due to disease progression); sorafenib was the second-line therapy (at 0.3 months treatment was terminated due to an adverse event), and re-challenging with sunitinib was the fifth-line therapy (at 4.6 months treatment was terminated due to disease progression).

Seven patients received IoT beyond third-line therapy. Three patients among these showed partial response (PR) and were alive at database lock (Table 2). No patient underwent cabozantinib or lenvatinib therapy (Supplementary Table 1).

### PFS and OS compared for each sequence until third-line treatment

To investigate the optimal sequence until the third-line treatment, we analysed the sequence types. Major sequences in the present cohort were αVEGF-αVEGF-αVEGF (17, 32.7%) and αVEGF-αVEGF-mTORI (12, 23.1%) (Supplementary Table 3). Other sequence types were excluded from the following analyses because of the low frequency. The median PFS of the third-line therapy with αVEGF-αVEGF-αVEGF was longer than that with αVEGF-αVEGF-mTORI (4.8 months vs. 2.0 months, respectively, p = 0.094) (Fig. 2C). The median OS from mRCC diagnosis in αVEGF-αVEGF-αVEGF was also relatively longer (60.1 months vs. 34.8 months, respectively); however, there was no statistical difference (p = 0.235) (Fig. 2D).

### Characteristics of the 103 patients who died during the follow-up period

To assess for the correlation between OS and the number of treatment lines administered during a patient’s lifetime, we extracted data on 103 patients who were deceased at database lock. Although the distributions by age and sex were similar between the 143-patient cohort and the 103-deceased-patient cohort, the deceased-patient cohort included a less favourable risk group (5.7% vs. 14.0%, respectively) and shorter median OS (17.7 months vs. 34.8 months, respectively) than the overall patient cohort (Table 1).

The rates of αVEGF/mTORI in the first-, second-, third-, and fourth-line therapies were 90.3%/8.7%, 80.3%/14.1%, 48.7%/33.3%, and 61.1%/33.3%, respectively, which were slightly dissimilar to those of the overall patient cohort. Three patients with PR using IoT were excluded from the deceased-patient cohort, because they were all alive at database lock.

Due to missing data, we could not obtain the MSKCC and IMDC criteria scores for 16 and 29 patients, respectively. Distribution between MSKCC and IMDC criteria showed strong correlation (Spearman correlation rate; 0.808), and there were more missing data among IMDC scores than among MSKCC scores. Hence, MSKCC criteria were adopted for the following analyses.

### Univariate analyses of the 103 patients who died during the follow-up period

Figure 2D shows the OS for each number of treatment lines administered during a patient’s lifetime. Although patients with triple treatment lines had longer survival rates than did patients with single- or double-line treatments, there was no significant difference when compared to patients that received quadruple or more lines of treatment (median OS: 42.5 months vs. 48.4 months, respectively, p = 0.422) (Fig. 2E). Univariate analyses showed statistically significant differences between the following: “single or double lines of treatment” and “triple or more lines of treatment” (p < 0.001), and between “triple or fewer lines of treatment” and “quadruple or more lines of treatment” (p = 0.001) (Table 3). Furthermore, we compared age (≤70 years vs. >70 years), sex, cT stage at RCC diagnosis, MSKCC criteria, surgery for the primary lesion, and metastatic site by univariate analysis. The parameters found to be significant for worse prognoses included worse MSKCC criteria, no nephrectomy, retroperitoneal lymph node metastasis, and central nerve system (CNS) metastasis (Table 3).

**Table 3:** Univariate analyses for overall survival (N = 103).

Factor	N (%)	Median OS (95% CI)	P-value
All	103	17.7 (14.2–29.2)	
Age			
≤70 years	67 (65.4)	19.2	0.739
>70 years	36 (37.6)	16.1	
Sex			0.59
male	79 (81.1)	22.2 (14.7–34.6)	
female	24 (21.9)	12.8 (9.8–25.0)	
cT stage at RCC diagnosis			0.097
1a	5 (4.2)	4.5 (3.8-NA)	
1b	20 (19.6)	22.0 (10.2–45.8)	
2a	11 (10.8)	46.8 (15.0–91.9)	
2b	5 (4.2)	10.4 (2.1-NA)	
3a	32 (31.4)	16.0 (10.0–32.2)	
3b	17 (16.7)	17.1 (8.3–40.9)	
3c	1 (1.0)	14.2 (NA-NA)	
4	11 (10.8)	11.1 (4.0–33.7)	
Unknown	1		
MSKCC criteria			<0.001
Favourable	5 (5.7)	15.0 (7.8-NA)	
Intermediate	61 (70.1)	22.2 (14.3–32.2)	
Poor	21 (24.1)	9.6 (4.5–10.5)	
Unknown	16		
Nephrectomy			<0.001
Yes	39 (37.9)	10.0 (7.6–12.4)	
No	64 (62.1)	33.3 (20.7–46.3)	
Pulmonary metastasis			
Yes	62 (60.2)	16.5 (12.2–32.2)	
No	41 (39.8)	22.2 (13.2–41.3)	
Osseous metastasis			0.887
Yes	37 (35.9)	15.2 (10.4–34.8)	
No	66 (64.1)	21.3 (14.2–32.2)	
Retroperitoneal lymph node metastasis			<0.001
Yes	26 (25.2)	10.8 (8.3–14.3)	
No	77 (74.8)	29.2 (16.3–40.9)	
Mediastinal metastasis			0.100
Yes	27 (26.2)	10.5 (8.3–17.7)	
No	76 (73.8)	25.6 (14.7–38.4)	
Hepatic metastasis			0.508
Yes	15 (14.6)	13.2 (7.3–22.2)	
No	88 (85.4)	21.3 (14.3–32.2)	
CNS metastasis			0.012
Yes	10 (10.0)	11.0 (3.6–14.3)	
No	93 (90.0)	21.8 (14.7–33.2)	
Number of treatment lines administered during patients’ lifetimes			
1	32 (31.1)	10.5 (8.3–20.7)	
2	32 (31.1)	13.5 (9.4–15.2)	
3	21 (20.4)	42.5 (14.2–53.4)	0.422
≥4	18 (17.4)	48.4 (32.2–61.6)	
≤2	64 (62.1)	11.6 (9.9–15.0)	<0.001
≥3	39 (37.9)	44.8 (33.2–56.5)	
≤3	85 (82.6)	14.3 (10.5–20.7)	0.001
≥4	18 (17.4)	48.4 (32.2–61.6)	

Abbreviations: N; number, OS; overall survival; 95% CI; 95% confidential interval, RCC; Renal Cell Carcinoma, MSKCC; Memorial Sloan Kettering Cancer Center, CNS; central nerve system.

### Multivariate analyses of the 103 patients who died during the follow-up period

We conducted two multivariate analyses. First, analysed data included “triple or more lines of treatment” along with the following parameters found to be prognostic factors by univariate analyses: MSKCC criteria, nephrectomy, retroperitoneal lymph node metastasis, and CNS metastasis. In this multivariate model, the independent prognostic factors were MSKCC criteria, nephrectomy, and patients with triple or more lines of treatment. Next, we analysed data that included “quadruple or more lines of treatment” instead of “triple or more lines of treatment” by multivariate analysis. “Quadruple or more lines of treatment” was not found to be an independent prognostic factor in this analysis (Table 4). These results implied that the fourth line does not have an impact on OS, while the third line contributes to improved OS.

**Table 4:** Multivariate analyses.

Analysed factors	HR (95% CI)
Worse MSKCC* criteria	1.932 (1.117–3.342)
No nephrectomy	2.525 (1.575–4.049)
**Retroperitoneal lymph node metastasis**	
CNS metastasis	
**Patients with ≥ 3 lines of treatment**	**0**.**368 (0**.**222–0**.**600)**
Worse MSKCC* criteria	2.549 (1.506–4.317)
No nephrectomy	2.237 (2.093–3.521)
**Retroperitoneal lymph node metastasis**	
CNS metastasis	2.394 (1.200–4.778)
**Patients with ≥ 4 lines of treatment**	

*MSKCC: Memorial Sloan Kettering Cancer Center; HR: hazard ratio.

## Discussion

With the increasing number of agents for mRCC having been developed since the 2000s, the efficacy of sequential therapy has been demonstrated. The RECORD-I study, a randomised phase III trial of everolimus versus placebo, first demonstrated the superiority of experimental agents for TT-refractory disease^14^. Axitinib yielded better PFS than did sorafenib^15^. As reported, lenvatinib plus everolimus prolonged PFS as compared to everolimus alone^16^. Nivolumab was the first agent to demonstrate a superior effect on OS as sequential therapy^7^. More recently, cabozantinib also demonstrated an OS benefit in an interim analysis^17^. Although there has been much evidence that sequential therapy improves survival, these improvements have been are mainly focused on second-line therapy results. Hence, high-level evidence that third- and fourth-line treatments improve survival is limited. Some retrospective studies have reported on third-line therapy; however, there have been no large-scale studies conducted for fourth-line therapy.

Prior retrospective studies and our study indicate that third-line therapy improves survival. In a subgroup analysis in RECORD-1 (an RCT comparing everolimus and placebo as sequential therapy), patients with third-line everolimus therapy showed longer PFS than did those with placebo (4.0 vs 1.8 months, respectively, p < 0.001)^14^. The IMDC group published a large-scale retrospective study for third-line TT. The median OS from cessation of second-line TT in those receiving third-line TT was 14 months, which was longer than the 2.1 months seen for those that did not receive third-line therapy^11^. Another Swiss single centre study reported that the median OS from the date of mRCC diagnosis until death in patients with equal or more than triple-line treatments was 43.8 months. This was longer than the 17.6 months for those with single- or double-line therapy^12^. In our study, the median OS was 44.8 months, which is comparable to the Swiss study results (Table 3 and Fig. 2A). The multivariate analysis showed that “triple or more lines of treatment” in patients was an independent prognostic factor for OS along with MSKCC criteria and prior nephrectomy (Table 4). Furthermore, approximately 20% of patients with third-line therapy had a long PFS of >15 months, as did those with second-line therapy (Fig. 2A,B). These results indicate that third line treatment could improve OS.

The optimal sequence for improving survival is unknown. In only one RCT, which compared temsirolimus and sorafenib as second-line therapies after sunitinib, the PFS in the sorafenib arm was relatively shorter (3.9 vs. 4.3 months, respectively, p = 0.19), while the OS was significantly longer (16.6 vs. 12.3 months, respectively, p = 0.01) than that in the temsirolimus arm^18^. Although the reasons for the contradiction between the PFS and OS are not fully understood, this RCT appears to show that second-line αVEGF therapy is superior to mTORI. However, some retrospective studies have reported different results than those of the RCT. Heng *et al*. reported a systematic review of second-line treatment, which analysed four retrospective multi-centre studies consisting of 1464 patients after αVEGF. They concluded that second-line mTORI therapy significantly prolonged survival when compared with second-line αVEGF therapy (HR = 0.82, 95% CI: 0.68–0.98)^19^. In our study, since only 5.8% of patients underwent a sequence using αVEGF-mTORI, we were unable to investigate the optimal second-line regimen. On the other hand, we were able to compare the third-line regimen after αVEGF-αVEGF (Supplementary Table 3). The results showed that the median PFS and OS in patients administered αVEGF-αVEGF-αVEGF were relatively longer than those administered αVEGF-αVEGF-mTORI (median PFS: 4.8 vs. 2.0 months, resepectively, p = 0.094; median OS: 60.1 months vs. 34.8 months, respectively, p = 0.235). Although our cohort was too small to elucidate the optimal sequence clearly, our findings might indicate that αVEGF should precede mTORI until the third-line treatment.

In fourth-line therapy, “quadruple or more lines of treatment” was not found to be an independent prognostic factor in our study. This result indicates that fourth line TT does not improve OS. To the best our knowledge, this is the first study to include the fourth-line therapy for OS. Previously, the Swiss group reported that 20.4% of patients (13 of 64) were treated with fourth-line therapy, and the RR evaluated by Response Evaluation Criteria in Solid Tumour (RECIST) version 1.0 was 15.4%^12^. In our study, almost the same number of patients (26 of 111, 23.4%) were treated with fourth-line therapy; however, the RR was 3.8%, which was less than that reported in the Swiss study. Although the reason for this difference is not understood, a possible reason is the difference in the RECIST versions that were used. We used RECIST version 1.1, which requires a pair of continuous responses to determine the clinical response. Hence, RECIST version 1.1 leads to less RR but reflects survival more precisely than version 1.0^12^. Actually, there were patients with temporary shrinkage who were no longer responding in our cohort. Therefore we suggest that rare patients have long efficacy leading to TT beyond three lines of therapy. Another Japanese group showed that the median PFS was 2.5 months, which was almost the same duration as that seen in our study. On the other hand, some patients with IoT showed clinical response even beyond third-line treatment (Table 2). Although our results of multivariate analysis indicated that the fourth-line treatments did not improve the OS, the analysed patients did not include clinical responders to IoT. Therefore, it is necessary to further investigate the efficacy of the latest agents including IoT.

Our study had several limitations. The most important one is the lack of data on the novel agents, including IoT, cabozantinib, and lenvatinib. Some patients had an objective response after third-line therapy due to IoT; however, they were not considered for the OS analysis, because patients who benefited from IoT were alive at database lock. However, the administration of novel agents was suggested to prolong the OS, even in fourth-line therapy. Second, it was a retrospective study. Receipt of three or more lines of treatment was an independent prognostic factor. However, this does not definitively indicate that third-line treatment prolongs survival, because the patients with longer survival should have received more treatment. Nevertheless, patients with third- and fourth-line treatments had almost the same survival rates. This could mean that fourth-line treatment does not prolong survival. Third, some of the patients lacked data with which to calculate MSKCC scores. Lastly, the study was based on a small cohort at a single centre.

In summary, third-line systemic therapy might improve OS, but fourth-line therapy does not in the absence of recent agents, such as IoT, cabozantinib, and lenvatinib.

## Methods

### Patients and methods

We retrospectively analysed 143 patients who started systemic therapies for mRCC in Yamagata University Hospital from January 2008 until December 2016. The last follow-up data were collected on 15 February 2018. Patients with previous cytokine therapy were allowed into this study; however, previous cytokine therapy was not counted as a therapeutic line. Patients who received only cytokine therapy were, therefore, excluded.

First, patients were analysed for the best objective response (BOR), RR, DCR, and median PFS for each line. Then, the BOR and median PFS were also determined for treated drug types for each line. The treated drug types were categorised into αVEGF, mTORI, IoT, and others. Furthermore, αVEGF and mTORI were defined as TT. αVEGF included sunitinib, sorafenib, pazopanib, and axitinib. mTORI included everolimus and temsirolimus. IoT included nivolumab.

To investigate an optimal sequence until third-line therapy, PFS and OS in third-line therapy were compared for each sequence. OS was calculated from the date of mRCC diagnosis until death or the last follow-up date. PFS and OS were compared using the log-rank test, with a significance level of 0.1.

To elucidate the correlation between OS and the number of regimens administered during the patients’ lifetimes, we extracted data on 103 mRCC patients who died during the follow-up period. OS, which was calculated from the date of mRCC diagnosis until death, was compared by the number of treatment lines. Furthermore, parameters, including age (≤70 vs. >70 years), sex (male vs. female), cT stage at RCC diagnosis, MSKCC criteria, IMDC criteria, nephrectomy, and metastatic site, were compared to estimate their possibilities as prognostic factors. Univariate analyses were conducted using the log-rank test, with a significance level of 0.05. Next, the number of treatment lines and factors that significantly indicated prognosis by univariate analyses were included in the multivariate analyses. The multivariate analyses using the Cox proportional model were built using a stepwise method, with a significance level of <0.05 for inclusion or exclusion of variables.

All parameters were assessed at the time of initial systemic treatment. BOR, RR, and PFS were basically determined by the RECIST version 1.1^20^. Complete response (CR), PR, or stable disease (SD) in the BOR decision was confirmed by a pair of continuous computerised tomography scans taken at greater than 1-month intervals. RR was calculated by the rate of CR and PR. DCR was calculated by the rate of CR, PR, and SD. When metastasectomy was performed without determining the progression of disease (PD) during medication, the patients were still included in PFS estimations, while the removal lesions were excluded from the target lesion. Furthermore, when patients undergoing radiotherapy had no target lesions, such as osseous metastasis, they were also included in the PFS analyses; however, they were excluded from the estimation of BOR. PFS, and OS were estimated by the Kaplan-Meier method. MSKCC and IMDC criteria were determined according to reports proposed by Motzer *et al*.^21^ and Heng *et al*.^22^, respectively. All statistical analyses were performed using the statistical software package R version 3.3.1.

The study was approved by the Ethics Committee of Yamagata University Faculty of Medicine (approval no. H30-534). The methods were carried out in accordance with the approved guidelines. The need for consent to participate in this study was waived by the same institutional review board.

## Supplementary information

Supplementary Tables
