# Supplementary material for: Effect of third- and fourth-line systemic therapies for metastatic renal cell carcinoma
Source: Sci Rep. 2019 Oct 29;9:15451. doi: 10.1038/s41598-019-51305-7 (PMC6820538; doi:10.1038/s41598-019-51305-7)
Supplement: Supplementary file 1 — Supplementary Tables [file 41598_2019_51305_MOESM1_ESM.pdf]

Effect of third- and fourth-line systemic therapies for metastatic renal cell carcinoma

Sei Naito*, Osamu Ichiyanagi, Tomoyuki Kato, Hidenori Kanno, Takafumi Narisawa, Masayuki Kurokawa, Masaki Ushijima, Michinobu Ozawa, Mayu Yagi, Yuta Kurota, Hiroki Fukuhara, Atsushi Yamagishi, Toshihiko Sakurai, Hayato Nishida, Hisashi Kawazoe, Takuya Yamanobe, and Norihiko Tsuchiya.

Department of Urology, Yamagata University Faculty of Medicine, Iida-nishi 2-2-2, Yamagata, 990-9585, Japan.

Tel: +81-23-628-5368; Fax: +81-23-628-5370

*Corresponding author: e-mail: seinaitoh@ybb.ne.jp

Running title: 3rd- and 4th-line therapies for mRCC

Supplementary Table 1. Number each treatment each line

	1st line		2nd line		3rd line		4th line		5th line		6th line		7th line		8th line	
	All	Patients who died	All	Patients who died	All	Patients who died	All	Patients who died	All	Patients who died	All	Patients who died	All	Patients who died	All	Patients who died
All	143	103	95	71	52	39	26	18	15	10	5	4	3	2	1	1
αVEGF	126	93	76	57	24	19	14	11	5	4	5	4	1	1	1	1
sunitinib	70	53	22	18	5	5	6	6	1	1	1	1	1	1	0	
sorafenib	31	28	18	13	10	8	3	1	1	1	0		0		0	
axitinib	19	10	34	25	6	4	4	3	1	1	2	2	0		1	1
pazopanib	6	2	2	1	3	2	1	1	2	1	2	1	0		0	
mTORI	9	9	12	10	15	13	7	6	3	3	0		0		0	
temsirolimus	8	8	3	3	2	2	1	1	3	3	0		0		0	
everolimus	1	1	9	7	13	11	6	5	0		0		0		0	
IoT	1	0	2	0	9	5	2	1	4	1	0		1	0	0	
nivolumab	0		2	0	9	5	2	1	4	1	0		1	0	0	
nivolumab+ipilimumab	1	0	0		0		0		0		0		0		0	
other	7	5	5	4	4	3	3	2	3	2	0		1	1	0	
TS-1	2	2	2	1	0		2	1	3	2	0		1	1	0	
UFT	0		1	1	3	3	0		0		0		0		0	
dovitinib	0		0		1	0	0		0		0		0		0	
CDDP based chemotherapy	2	2	1	1	0		1	1	0		0		0		0	
Ca based chemotherapy	0		1	1	0		0		0		0		0		0	
TS-1+sorafenib	2	1	0		0		0		0		0		0		0	
avelumab+axitinib	1	0	0		0		0		0		0		0		0	

Supplementary Table 2. Duration and PFS each drug type each line

	1st line			2nd line			3rd line			4th line			5th line			6th line			7th line			8th line		
	N	DURATION*	PFS**	N	DURATION*	PFS**	N	DURATION*	PFS**	N	DURATION*	PFS**	N	DURATION*	PFS**	N	DURATION	PFS**	N	DURATION*	PFS**	N	DURATION*	PFS**
αVEGF	126	4.5 (0.1-43.5)	6.2 (4.1-8.8)	76	2.2 (0.1-75.8)	2.5 (1.0-5.7)	24	2.9 (0.2-49.8)	4.1 (2.2-18.9)	14	2.4 (0.9-9.2)	3.7 (1.4-7.6)	5	2.1 (1.1-6.2)	3.4 (1.8-NA)	5	4.6 (0.6-18.6)	4.6 (0.6-NA)	1	2.1	2.1	1	3.0	3.0
mTORI	9	1.1 (0.1-4.2)	1.4 (0.1-2.1)	12	1.9 (0.2-54.3)	3.5 (1.0-NA))	15	1.8 (0.4-23.9)	2.0 (0.8-4.2)	7	5.4 (0.7-15.0)	5.4 (0.7-NA)	3	1.6 (1.6-6.7)	1.6 (1.6-NA)	0			0			0		
IoT	1	5.5	NA	2	1.84	NA	9	3.1 (0.7-8.7)	3.7 (0.7-5.5)	2	4.0 (3.4-4.7)	4.0 (3.4-NA)	4	4.3 (0.2-11.7)	0.7 (0.2-NA)	0			1	7.4	7.4	0		
other	7	2.5 (1.6-26.2)	2.5 (1.6-19.5)	5	71.2 (2.5-140.1)	2.5 (2.5-NA)	4	2.4 (1.1-2.6)	2.4 (1.1-NA)	3	2.3 (0.5-5.1)	5.1 (0.5-NA)	3	1.2 (0.8-3.6)	1.3 (0.9-NA)	0			1	0.4	0.4	0		

	1st line			2nd line			3rd line			4th line			5th line			6th line			7th line			8th line		
	N	DURATION*	PFS**	N	DURATION*	PFS**	N	DURATION*	PFS**	N	DURATION*	PFS**	N	DURATION*	PFS**	N	DURATION	PFS**	N	DURATION*	PFS**	N	DURATION*	PFS**
αVEGF	93	3.2 (0.1-43.5)	4.7 (2.8-8.7)	57	1.5 (0.1-15.2)	1.8 (0.9-2.9)	19	2.5 (0.2-38.4)	3.2 (1.4-4.8)	11	2.4 (0.9-7.8)	3.7 (0.9-7.6)	4	3.2 (1.1-6.2)	4.6 (1.8-NA)	4	4.6 (0.6-18.6)	4.6 (0.6-NA)	1	2.1	2.1	1	3.0	3.0
mTORI	9	1.1 (0.1-4.2)	1.4 (0.1-2.1)	10	1.9 (0.2-10.6)	1.9 (1.0-NA)	13	1.3 (0.4-12.4)	1.3 (0.7-3.7)	6	2.1 (0.7-15.0)	2.1 (0.7-NA)	3	1.6 (1.6-6.7)	1.6 (1.6-NA)	0			0			0		
IoT	0			0			5	1.8 (0.7-5.5)	3.7 (0.7-NA)	1	3.4	3.4	1	0.2	0.2	0			0			0		
other	5	2.5 (1.6-19.5)	2.5 (1.6-NA)	4	2.5	2.5 (NA-NA)	3	2.5 (2.5-2.6)	2.5 (2.5-NA)	2	2.8 (0.5-5.1)	2.8 (0.5-NA)	2	2.3 (0.9-3.6)	2.3 (0.9-NA)	0			1	0.4	0.4	0		

*DURATION; duration from start of treatment to date of progression or last follow-up, **PFS; progression free survival

Supplementary Table 3. Sequence until third-line therapy

sequence	N	%
αVEGF/αVEGF/αVEGF	17	32.7
αVEGF/αVEGF/mTORI	12	23.1
αVEGF/αVEGF/IoT	9	17.3
αVEGF/αVEGF/other	3	5.8
αVEGF/mTORI/αVEGF	1	1.9
αVEGF/mTORI/mTORI	1	1.9
αVEGF/mTORI/other	1	1.9
mTORI/αVEGF/αVEGF	1	1.9
other/αVEGF/αVEGF	2	3.8
other sequence	5	9.6
